# Primary Invasive Paget’s Disease in a Clinical Practice: A Case Report

**DOI:** 10.7759/cureus.61381

**Published:** 2024-05-30

**Authors:** Timia Raven-Gregg, Sharat Chopra, Prav Hamal

**Affiliations:** 1 General Surgery, Aneurin Bevan University Health Board, Newport, GBR; 2 Emergency Medicine, University Hospital of Wales, Cardiff, GBR

**Keywords:** breast cancer pathology, primary breast malignancy, invasive paget's disease, general surgery and breast cancer, oncoplastic breast surgery, paget's disease of the breast

## Abstract

Invasive Paget’s disease (IPDN) is a rare phenomenon characterised by invasive carcinoma localised to the nipple. It is associated with Paget’s disease of the nipple (PDN) whereby Paget cells locally invade the underlying epidermis. Often in PDN, histopathological confirmation is needed, due to a lack of clear symptoms on clinical presentation. An 82-year-old female with single duct ectasia presented to the breast clinic in September 2023 with a tender, inflamed right nipple with a necrotic appearance. The lesion was excised, and an ultrasound scan showed right U2, implying no underlying malignancy. Microscopy showed Paget’s disease with underlying ductal carcinoma in situ and two small (0.4 and 0.3mm) foci of dermal invasion by Paget cells in keeping with IPDN. Research suggests that dermal invasion by Paget cells has little effect on clinical outcome and prognosis depends largely on the associated underlying malignancy. However, all cases of IPDN with deep invasion or penetration of Paget cells into the dermis have the potential for regional and distant lymphatic spread. In extramammary Paget’s disease, depth of invasion has been associated with poorer survival. Therefore, wide variability in clinical patterns and presentations of PDN mandates that a careful clinical approach correlated with in-depth histopathological evaluation is adopted in all cases.

## Introduction

Paget’s disease of the nipple (PDN) is an intraepithelial neoplasm described by Sir James Paget in 1874 [1]. Clinically, PDN is characterised by an eczematous or ulcerative lesion over the nipple. It typically presents as a unilateral erythematous eruption with a scaly crust causing pruritis. In around 50% to 60% of women with PDN, an underlying mass is found which usually signifies an underlying invasive carcinoma [2,3]. Despite this, PDN is considered a rare manifestation of breast cancer, with an incidence of 0.7% to 4.3% across all cases [4,5].

There are three types of PDN recognised according to pathophysiology: classical, secondary and invasive Paget’s disease (IPDN). In classical PDN, the carcinoma cells migrate along lactiferous ducts to involve the nipple epidermis. Tumour cells disrupt the normal epithelial barrier, allowing extracellular fluid to seep into the epithelial surface. This involves an intraductal or underlying invasive carcinoma. Secondary PDN occurs when the underlying invasive carcinoma extends directly into the nipple epidermis. Carcinoma cells extend via the basal layer, penetrating through the epidermis. These tumours are often T4d staged. IPDN, however, involves invasive carcinoma localised to the nipple, pathognomonic of PDN. There may also be underlying intraductal or invasive carcinoma of the underlying breast. There are two theories proposing a mechanism behind IPDN. These are the epidermotropic and transformation theories. The epidermotropic theory suggests that there is migration of Paget's cells from underlying carcinoma into the nipple epidermis. The transformation theory suggests that there is a malignant transformation of the cells within the nipple epidermis [6]. As a result, most cases of IPDN are T1mi (microinvasion) or T1a [7]. Therefore, in IPDN, T staging is often variable (see Table 1 in Appendices for TNM staging of breast cancer). The incidence of IPDN in all cases of Paget’s disease is reported to be between 4% and 7.8%. In this case report, we will explore the implications of a case of IPDN in clinical practice relating it to the current literature.

## Case presentation

**Figure 1:**
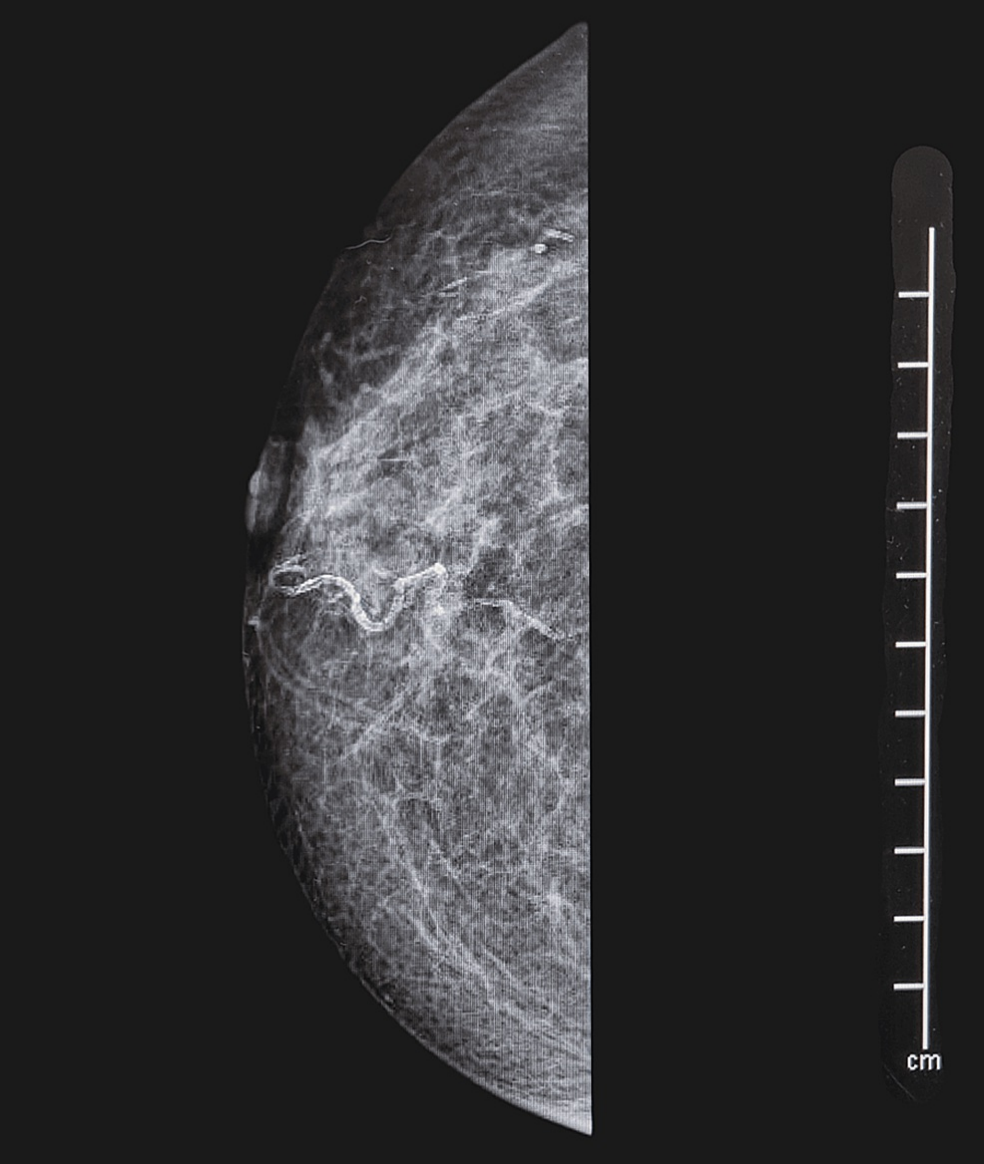
Mammogram (30 January 2019) of the right breast at first presentation showing slight nipple inversion with increased echogenic material in the lactiferous ducts.

In 2019, an 82-year-old female presented to her general practitioner with a history of blood-stained discharge from the right nipple for three months. She had no other concerning features for breast cancer at the time. She had a past medical history of diverticular disease, chronic obstructive pulmonary disease, hypertension, celiac disease and osteoarthritis. She had no significant family history of breast or ovarian cancer. She underwent the menopause around age of 50 years and has three children all of which were breastfed. She had a two-month history of prior hormonal replacement therapy use three years ago and is an ex-smoker, with a 20-pack-year history. Examination in the clinic demonstrated easily elicitable nipple discharge from a single duct, with no palpable lumps in either breast or axillae. A subsequent mammogram demonstrated echogenic material within the lactiferous ducts (Figure 1).

**Figure 2:**
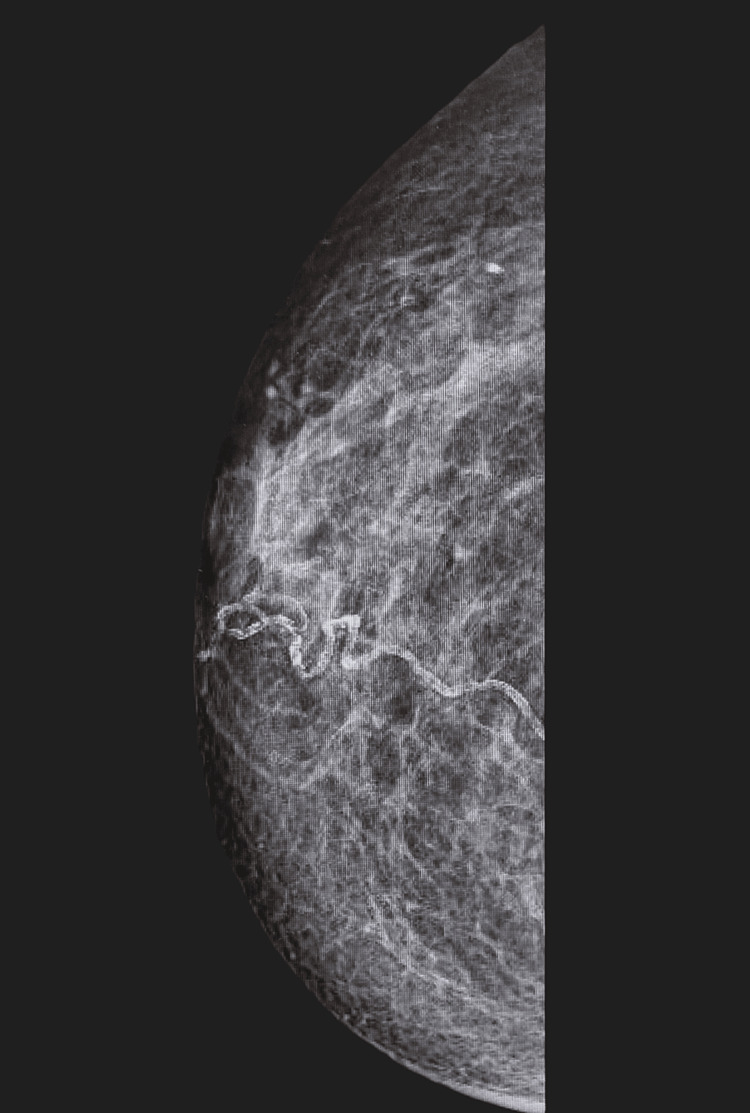
Mammogram (6 December 2021) of right breast showing more pronounced nipple inversion but no apparent underlying mass within the right breast tissue.

Whilst imaging was largely reassuring, it was recommended that a precautionary primary duct excision should be performed for further histological analysis. However, the patient declined. Following a repeat presentation in 2021 with the same symptoms, a mammogram and subsequent ultrasound scan were performed. Aside, from more pronounced nipple inversion, this was again largely reassuring, and the patient was discharged with a clinical diagnosis of primary duct ectasia (Figure 2).

**Figure 3:**
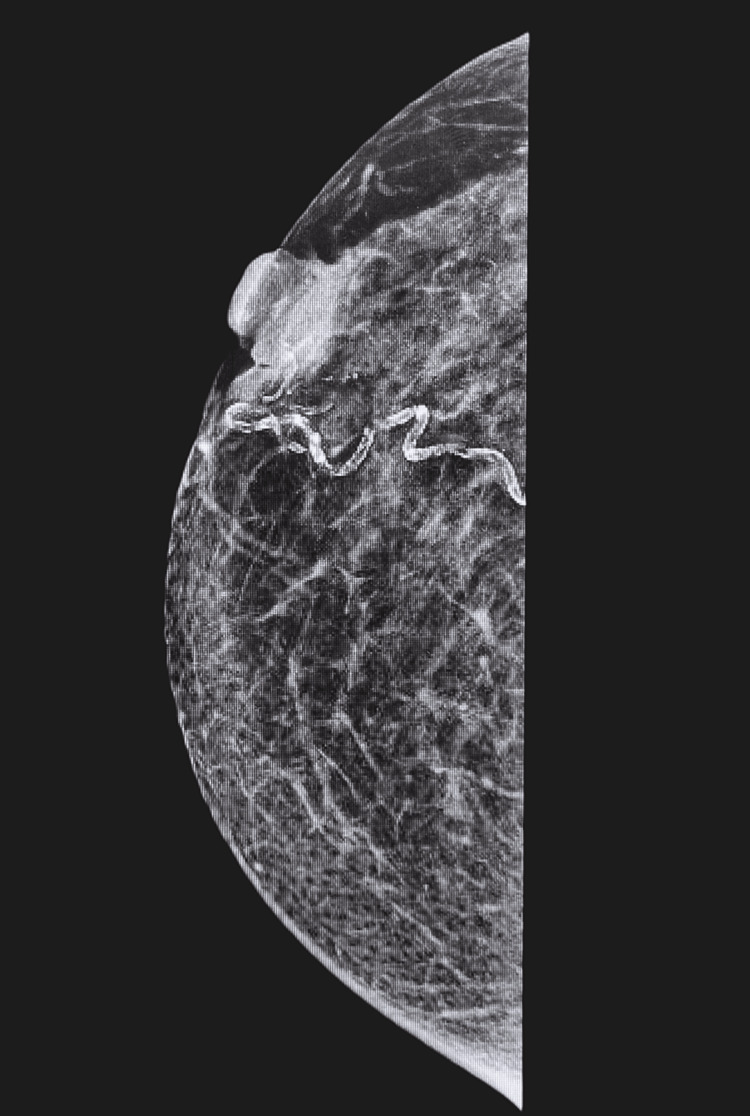
Mammogram (13 September 2023) of right breast showing underlying mass beneath nipple (M4).

A repeat presentation to the clinic in September 2023 revealed a progressively red, sore and inflamed right nipple. For the last few months, the patient had been suffering from increased production of exudate and bleeding from the nipple. Again, there was no palpable mass on examination. Mammography was performed and revealed an underlying 19mm mass, new since the prior mammogram in 2021 (Figure 3).

**Figure 4:**
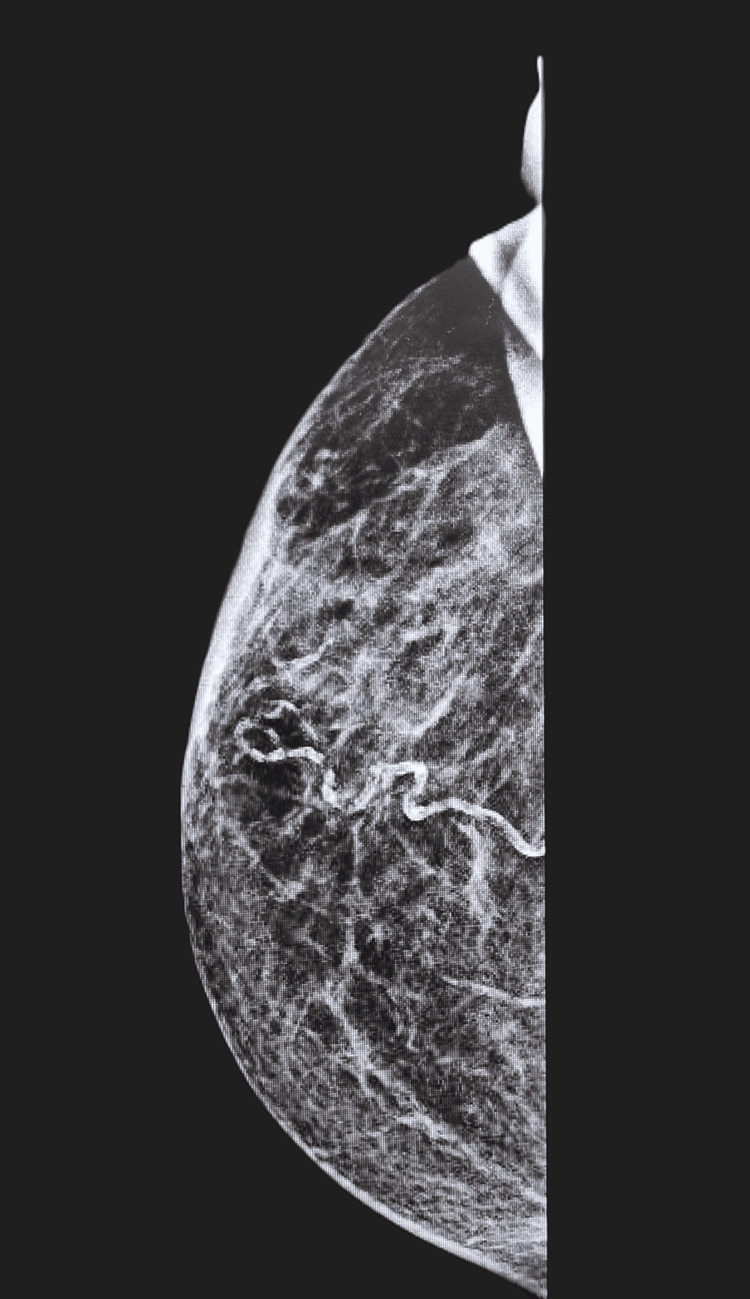
Mammogram (3 October 2023) of right breast showing no evidence of underlying mass after excision (U2).

The report stated: "Both breasts have a mixed fatty and glandular appearance (glandular density 25%). There is right nipple inversion, with an underlying 19mm mass which is new since the previous mammogram of 2021. This may represent a collection, but malignancy cannot be excluded. The left breast is unremarkable. Right breast M4, Left breast M2." The 2cm lesion appeared necrotic and clinically suggestive of papilloma. This was subsequently excised in the clinic and examined histologically. After excision, an ultrasound of the right breast showed no focal lesion or adverse radiological features, and no underlying mass within the breast tissue (Figure 4). The report stated: "It is understood that there was a lesion on the nipple which has been removed in the clinic and is considered to be the cause of the mammographic mass. Within the right subareolar region, scanning immediately beneath the nipple demonstrates dilated ducts, no focal lesion or adverse features identified. This is consistent with Right breast U2."

**Figure 5:**
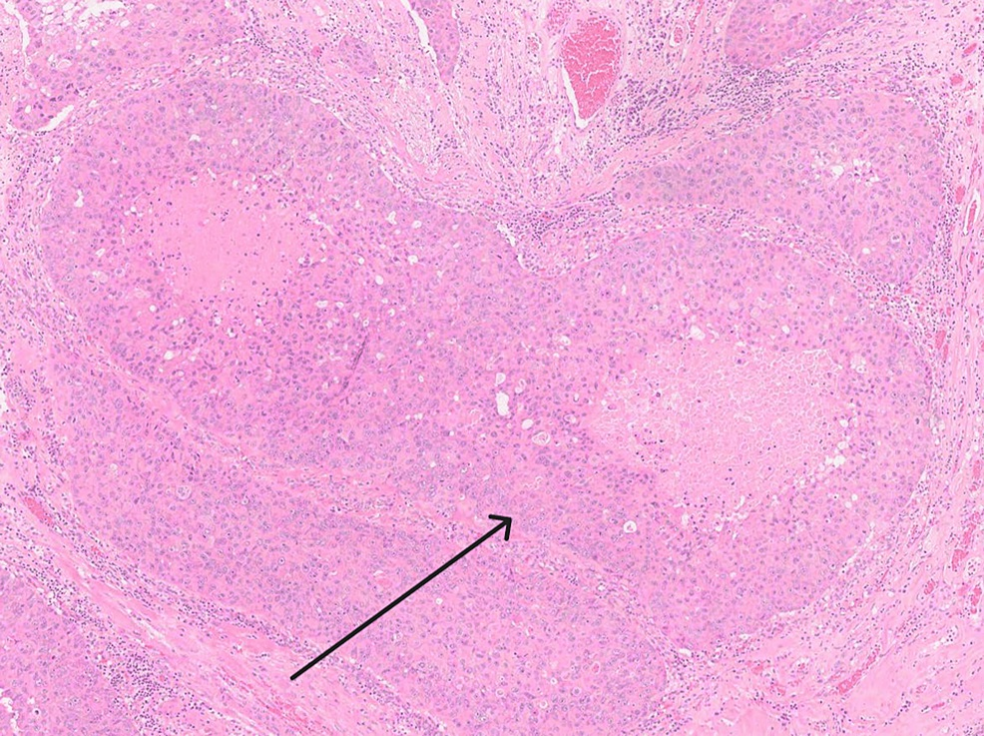
Histological specimen showing the area of underlying DCIS. DCIS: ductal carcinoma in situ

**Figure 6:**
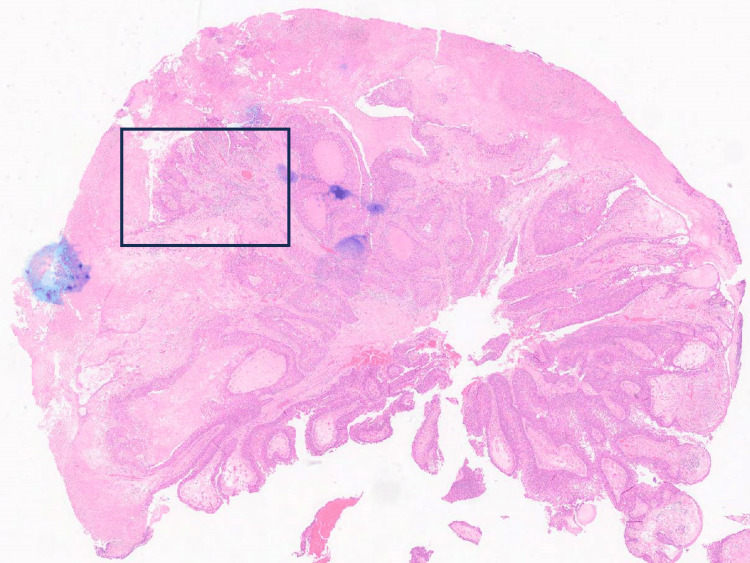
Histopathological specimen showing area of invasive Paget's disease.

**Figure 7:**
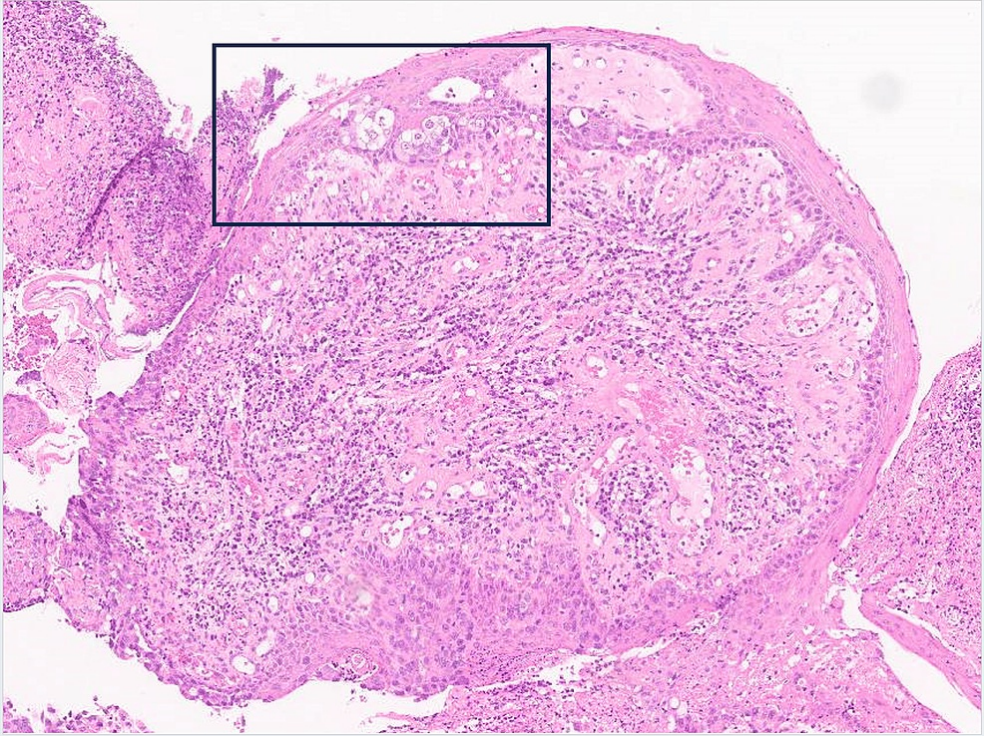
Histological slide demonstrating the area of infiltration of invasive Paget's disease with characteristic Paget's cells visible.

**Figure 8:**
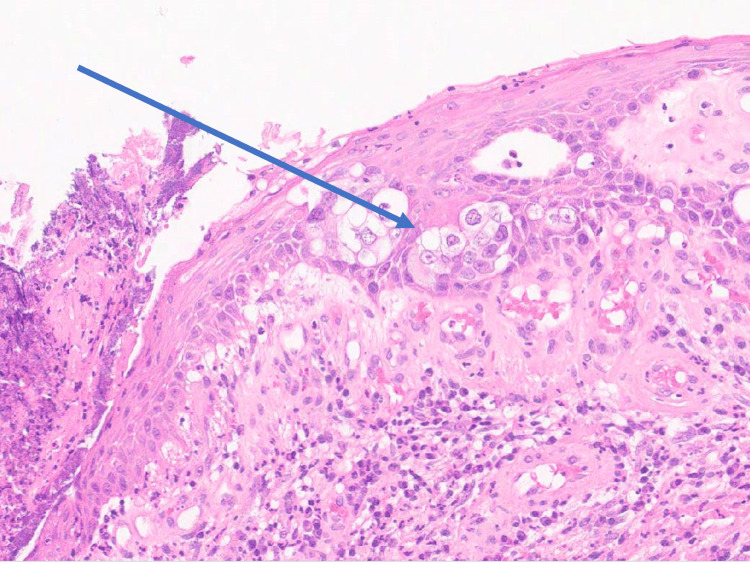
Histological slide showing a close-up of Paget cells characterised by large malignant cells with abundant cytoplasm.

Microscopy showed an ulcerated papillomatous lesion and florid Paget's disease (with underlying lactiferous sinuses involved by high-grade ductal carcinoma in situ) (Figure 5). There were two small (0.4mm and 0.3mm) foci of dermal invasion by Paget cells identified (Figures 6-8). In the absence of underlying invasive breast cancer, features were deemed consistent with primary invasive Paget disease (PT1a, lesion incompletely excised). The malignancy was oestrogen receptor (ER), progesterone receptor (PR) and Herceptin receptor (HER2) negative. The patient went on to have a total mastectomy with sentinel lymph node biopsy. The mastectomy specimen was examined grossly and subsequent histological section examination was performed which ruled out an invasive lesion in the breast. Lymph node biopsy revealed no evidence of malignant lymphatic spread. The patient did not require any hormonal treatment nor chemotherapy. At six-month follow-up, there is no evidence of recurrence and the patient has recovered well postoperatively.

## Discussion

In primary PDN, a nipple biopsy is diagnostic and shows shotgun infiltration of the epidermis with large malignant cells and abundant cytoplasm. Paget cells are HER2 positive in greater than 90% of cases with immunohistochemistry staining. The results of two studies suggest that most cases (80% to 96% of cases of PDN) show HER2 overexpression, in contrast to our case. Research suggests that so far, between 10% and 28% of IPDN cases are detected by histopathologic examination of the nipple in mastectomy specimens alone, in the absence of any apparent clinical abnormalities. This is consistent with our case, whereby following an excision biopsy in the clinic, the lesion was thought to be completely excised following ultrasound. Microscopic examination revealed that the lesion was incompletely excised. Another case reported in the literature demonstrated that a patient with right-sided breast cancer (thought to be Stage 1) treated with lumpectomy and axillary lymph node dissection, presented 20 years later with an ipsilateral nipple rash and evidence of microinvasion, characteristic of IPDN. In this case, the patient reported right nipple eczema and rash intermittently over several years. With limited information regarding the initial breast cancer, it is hard to say whether there could have been evidence of IPDN at the initial presentation which was incompletely excised. In the presence of ipsilateral axillary lymph nodes, this could have carried a risk for regional lymphatic involvement [8].

Two of the 35 cases of IPDN identified across the literature had less than 3.0mm depth of invasion [1,9-11]. Microinvasion (<1mm) as seen in our patient case is consistent with most cases reported across the literature. In 12.1% of IPDN cases, no underlying invasive or in situ carcinoma was identified. The most common presenting symptom was a scaly skin change overlying the nipple. In our case, the patient had an underlying ductal carcinoma in situ, whereas most symptomatic cases have typically been found to have an underlying invasive carcinoma. The age range of patients with IPDN is reported as between 35 and 76, making our patient the oldest case reported thus far [6]. Six of 35 patients had metastasis to regional lymph nodes at the time of their initial surgical procedure [1,9]. However, these patients also had an underlying invasive breast cancer. In one case of HER2 positive, ER negative and PR negative IPDN, a central lumpectomy was performed. The patient presented with ipsilateral palpable axillary lymphadenopathy three months later (seven of 19 nodes positive for lymphatic invasion) despite starting chemotherapy [2]. The invasive component was a 6mm mass underneath the nipple skin. In this case, it is likely that the tumour represents an invasive carcinoma with concurrent ductal carcinoma in situ and PDN, rather than IPDN alone. A positive HER2 receptor status usually implies a more aggressive clinical picture, however, in one reported case of HER2-negative IPDN, regional lymph node metastasis was present [1]. Therefore, the wide variability in the presentation and staging of IPDN mandates that we treat each case both individually and carefully [2]. Thus far, there is no report of local recurrence in IPDN cases across the literature [12]. Reports suggest that in cases of IPDN, the underlying carcinoma is most important, and dermal invasion by Paget cells has little effect on the prognosis [9,12,13].

Another condition, extramammary Paget’s disease (EMPD) is thought to behave similarly. The condition develops in apocrine gland-bearing areas and presents characteristically erythematous lesions with overlying crust and scale resembling disorders such as eczema, leading frequently to misdiagnosis [14]. Like IPDN, dermal invasion is an essential criterion for diagnosis [13]. In EMPD, a clear distinction needs to be made between primary and secondary infiltration, whereby the latter has a more indolent clinical course. Once Paget cells invade deeply into the dermis, regional and distant lymph node metastases are possible. In such cases, the prognosis is often poor.

## Conclusions

Clinical diagnosis of PDN is most likely underestimated and careful examination of histopathology is crucial in detecting IPDN cases. It is clear that there is a paucity of evidence on long-term outcomes with regard to recurrence in cases of IPDN. Whilst little is known about the behaviour and progression of IPDN, Paget cells themselves are characteristically known for their potential to rapidly spread. Evidence suggests that the invasive component of IPDN has little bearing on the overall prognosis. However, if there is a clinical correlation between IPDN with invasive EMPD, we should be treating all cases of PDN with more caution. Whilst most cases of confirmed IPDN appear to be microinvasive and respond well to treatment, there is potential for misdiagnoses of true clinical cases. In the event of IPDN with aggressive underlying malignancy, failure to detect cases could lead to poor patient outcomes. There is large variability in the clinical presentations and outcomes of classical, secondary and primary IPDN. Therefore, greater awareness and education surrounding the clinical presentations of EMPD and PDN are needed to avoid misdiagnoses, enable efficient and prompt treatment and allow for careful monitoring of disease progression.

## Appendices

**Table 1:** TNM staging of breast cancer (adapted from Hartmann-Johnsen 2019) [5].

Tumour	T0/Tis	T1	T2	T3	T4
Tumour Size	T0: No primary tumour Tis: tumour only in breast ducts or lobules	≤2cm	>2cm-≤5cm	>5cm	Tumour of any size with extension to chest wall/skin (ulceration or skin nodules)
Node	N0	N1	N2	N3	
	N0 lymph node metastases	Metastases in 1-3 axillary lymph nodes	Metastases in 4-9 axillary lymph nodes	Metastases in infra-or supraclavicular lymph nodes or in ≥ 10 axillary lymph nodes	
Metastasis	M0	M1			
	No evidence of cancer metastases	Distant metastasis			
